# A convenient protocol for generating giant unilamellar vesicles containing SNARE proteins using electroformation

**DOI:** 10.1038/s41598-018-27456-4

**Published:** 2018-06-21

**Authors:** Agata Witkowska, Lukasz Jablonski, Reinhard Jahn

**Affiliations:** 1 Department of Neurobiology, Max-Planck-Institute for Biophysical Chemistry, Göttingen, Germany; 2 Institute for Auditory Neuroscience, University Medical Center Göttingen, Göttingen, Germany; 3 Auditory Neuroscience and Optogenetics Laboratory, German Primate Center, Göttingen, Germany

## Abstract

Reconstitution of membrane proteins in artificial membranes is an essential prerequisite for functional studies that depend on the context of an intact membrane. While straight-forward protocols for reconstituting proteins in small unilamellar vesicles were developed many years ago, it is much more difficult to prepare large membranes containing membrane proteins at biologically relevant concentrations. Giant unilamellar vesicles (GUVs) represent a model system that is characterised by low curvature, controllable tension, and large surface that can be easily visualised with microscopy, but protein insertion is notoriously difficult. Here we describe a convenient method for efficient generation of GUVs containing functionally active SNARE proteins that govern exocytosis of synaptic vesicles. Preparation of proteo-GUVs requires a simple, in-house-built device, standard and inexpensive electronic equipment, and employs a straight-forward protocol that largely avoids damage of the proteins. The procedure allows upscaling and multiplexing, thus providing a platform for establishing and optimizing preparation of GUVs containing membrane proteins for a diverse array of applications.

## Introduction

Giant unilamellar vesicles (GUVs) represent artificial vesicles with a diameter of >1 µm. Originally introduced many years ago by Reeves and Dowben^1^, GUVs have only recently become more popular for studying the properties of membrane proteins^2^. Their main advantages are: (i) GUVs can be conveniently visualised with basic light microscopy techniques (in contrast to smaller vesicles); (ii) GUVs, even if immobilised, are largely free of curvature stress provided that the surface contact area is comparatively small; and (iii) GUVs can be easily manipulated, and their membrane tension can be precisely controlled. However, due to the large size of GUVs imaging of the membrane surface requires 3D scanning and thus limits the time resolution of the experiment. Moreover, preparation of GUVs with a desired protein and lipid composition can be challenging^3^: There is no universal protocol because different procedures may be required to achieve optimal results depending on, among other factors, protein, lipid, and buffer compositions.

Traditionally, GUVs are formed with a gentle hydration method^1^, but this method is rather slow and inefficient^3^. A major improvement was introduced by Angelova and Dimitrov^4^ with the electroformation technique. In this method, vesicles are formed from a dried lipid film while an external electric field is applied. Initially, lipid films were deposited directly on platinum (Pt) electrodes^4^. Later this technique was modified to work with glass slides coated with a conductive surface (indium tin oxide, ITO)^5^. Additional GUV preparation methods have also been developed including osmotic shock^6^, gel assisted swelling^7,8^, peptide-induced fusion of smaller precursor vesicles^9^, detergent-mediated reconstitution^10^, droplet-transfer method^11^, or an inkjet-based formation^12^.

Preparation of protein-containing GUVs (proteo-GUVs) has been a particularly challenging task due to the fact that GUV-formation usually includes steps that can denature proteins. Such steps may include: (i) formation of a lipid mixture from lipids diluted in organic solvents, (ii) drying, and (iii) formation of vesicles in a salt-free environment (but note that there are ongoing efforts to optimize protocols utilizing physiological ionic strength buffers, see e.g. ref.^13^). Despite these drawbacks, GUVs are increasingly used for *in vitro* reconstitution of membrane proteins and for the study of membrane remodelling, for instance for studying bacteriorhodopsin^14^, ion channels^13,15,16^, or exocytosis of synaptic vesicles^17–19^.

Nevertheless, the preparation of proteo-GUVs still requires time-consuming optimization as protocols are frequently difficult to reproduce between laboratories (own experience and personal communication with other researchers in the field), largely because not all variables affecting the outcome are controlled and optimised. This includes, for instance, design of the electroformation chamber including slide resistance when using ITO slides, spacer thickness (that is necessary for calculating the electric field), and for Pt electrodes information about the thickness and the axial distance between the wires. Moreover, the parameters of the applied electric field are critical for the outcome, with the results depending on the chamber geometry and the precise voltage-time profile of the applied electric field.

Here we report a convenient protocol for the preparation of proteo-GUVs containing functionally active neuronal SNARE (soluble N-ethylmaleimide-sensitive factor activating protein receptor) proteins for the study of membrane fusion *in vitro*. SNARE proteins represent a superfamily of small, mostly membrane-anchored proteins that catalyse the fusion of membranes in all eukaryotic cells. In neurons, exocytosis of synaptic vesicles is mediated by the SNARE proteins syntaxin-1A and SNAP-25 present at the plasma membrane, and synaptobrevin-2 present on the vesicles. Our protocol is straightforward and requires only a simple and affordable, in-house-built setup, therefore it can be easily adapted for other proteins and lipid compositions.

## Results

### Setup Design

**Figure 1:**
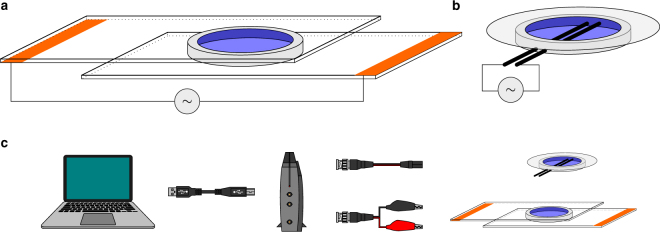
Setup for electroformation of GUVs. (**a**) Schematic representation of an electroformation chamber with ITO slides. SUVs are dried on the conducting surface of an ITO-coated glass slide, each containing a stripe of copper tape (orange stripes) attached to the slide with a conductive glue. The electroformation chamber is then assembled from two slides, with the conductive surfaces facing each other and held apart by a 3 mm-thick ring-shaped silicon spacer (light grey). The rehydration buffer (200 mM sucrose) is injected into the chamber with a syringe by puncturing the silicone spacer with a thin needle, and with a second needle positioned on the opposite side for air removal. The function generator (∼) is connected to the copper stripes with crocodile clips. Based on the original chamber design presented in ref.^5^ and used in ref.^18^. (**b**) Schematic illustration of a Pt chamber where SUVs are dried directly on the surface of Pt wires (black rods). (**c**) Equipment needed for GUV electroformation: computer, function generator, connecting wires, and electroformation chambers.

Electroformation of GUVs can be performed by applying an alternating electric field in the formation chamber that either consists of two glasses coated with conductive material (such as ITO) that are separated by a spacer (see e.g. refs^18,20–22^; Fig. 1a), or that contains two Pt electrodes (presented in this work; Fig. 1b). In the first approach, the parameters that critically influence the electroformation are the electrical resistance of the ITO coat and the distance between the two conductive surfaces. When Pt wires are used for preparation of GUVs, the two important parameters are the axial distance and the thickness of the two parallel electrodes. In both cases, additional parameters have a strong influence on GUV formation such as chamber volume, chamber cleanliness and deterioration due to repeated use, concentration of lipids, the method of lipid drying, and the composition of the buffer used for electroformation.

**Figure 2:**
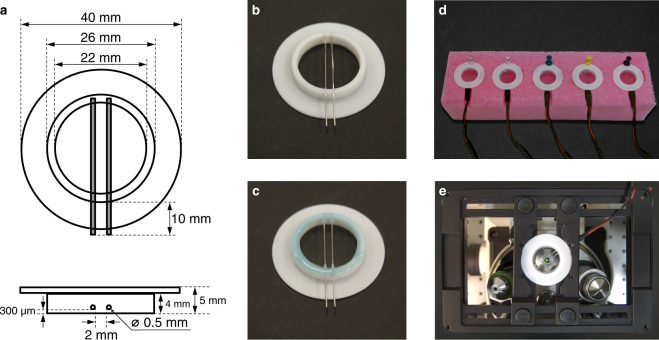
Design of the Pt chamber. (**a**) Technical drawing of the Pt chamber made from PTFE and containing platinum wires (grey). (**b** and **c**) Photographs of the electroformation chamber. In (**c**) the chamber is sealed with a coverslip. Chambers (**b** and **c**) are placed upside down for clarity. (**d** and **e**) Photographs of the chambers during the electroformation either on the lab bench in a polyethylene foam stand (**d**) or on a microscopy stage (**e**).

Previously, we prepared SNARE-containing GUVs using ITO-coated glasses^18,20^ (see also Fig. 1a). However, GUV formation was not easily reproducible and yielded relatively small GUVs (often below 5 µm in diameter), prompting us to work out a more reliable protocol that would yield larger GUVs. We therefore designed a Pt wire-based electroformation chamber (in short Pt chamber; Figs 1b and 2a; other designs are presented for example in refs^13,23^). The main idea was to design a chamber that could be effectively cleaned with organic solvents (to remove residual lipids), and that allows for easy monitoring of GUV formation (compatibility with a standard Zeiss microscopy stage). For this purpose, a largely chemically inert PTFE (polytetrafluoroethylene) was chosen as the main material for the chamber with two Pt wires (0.5 mm in diameter) embedded close to the chamber bottom. The dimensions of the chamber were chosen to allow for sealing with a standard size (25 mm in diameter) microscopic coverslip and to fit, together with the wiring, on a microscopy stage (Figs 1b and 2a,e). For electroformation we used a digital function generator (Velleman PCGU1000), connected via USB to a Windows PC (Fig. 1c). This function generator is inexpensive in comparison to other (usually stand-alone) laboratory function generators, and the output voltage waveform can be easily programmed within the accompanying software (PcLab2000SE, Velleman). The Pt chamber was connected to the function generator using cable with BNC connector and pin socket. In this setup socket pitch of 2.54 mm fits the 2.5 mm axial distance of Pt electrodes, while for the ITO chamber the latter were replaced with crocodile clips (Fig. 1c). Additionally, by using BNC Y-splitters, multiple chambers can be connected to one function generator. In conclusion, the whole electroformation setup consists of a PC, a function generator, connecting cables, and electroformation chambers (Fig. 1c). One electroformation chamber can be then placed on a microscope (as shown in Fig. 2e, to allow live monitoring, see Supplementary Video 1), while others can be placed for stability in a suitable stand (like the one made from the polyethylene foam shown in Fig. 2d).

### Formation of GUVs in a Pt chamber

**Figure 3:**
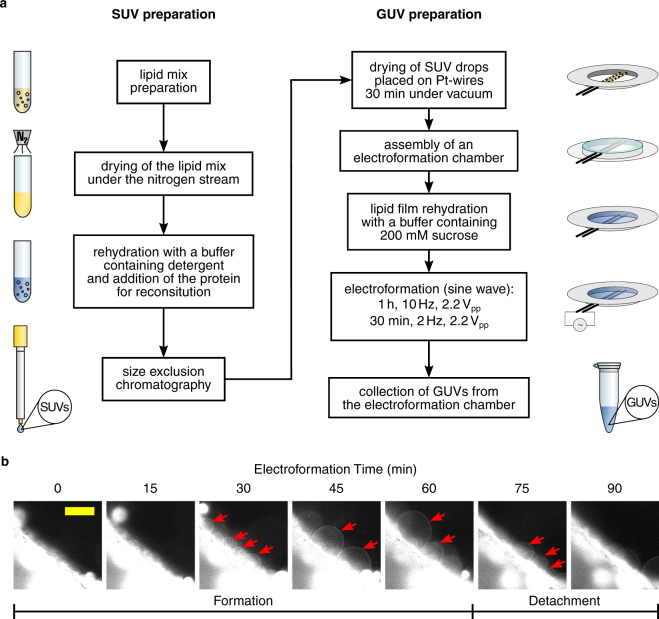
Overview over the steps required for forming proteo-GUVs. (**a**) Flowchart representing the workflow for preparation of protein-containing GUVs, starting from the preparation of SUVs. (**b**) Snapshots of the electroformation of SNARE-GUVs on a Pt wire. Membranes were labelled with the lipophilic dye DiO for fluorescent visualization. GUVs are indicated with red arrows. Scale bar 50 µm. See also Supplementary Video 1.

The most common approach for formation of GUVs containing transmembrane proteins is to start with small liposomes reconstituted with membrane proteins using standard protocols, e.g. using detergent removal by size exclusion chromatography^3,20,24^ (Fig. 3a). These proteoliposomes are deposited on the conductive surface in the electroformation chamber and dried in order to remove the aqueous buffer. For instance, for preparing SNARE-GUVs with a Pt chamber 5–7 × 1 µl drops of SUVs are deposited on each Pt wire (10–14 drops/Pt chamber) and dried under vacuum for around 30 minutes (Fig. 3a). Next, the Pt chamber is sealed with a coverslip (25 mm diameter, coated with β-Casein to prevent bursting of GUVs making contact with the glass surface) and a silicone glue (see photo in Fig. 2c). The sealed chamber is then connected to the function generator and filled with an electroformation solution — typically water with sucrose (we used 800 µl of 200 mM sucrose solution in each chamber; see photos in Fig. 2d and e). Immediately afterwards electroformation is started by switching on the AC field. In our hands, the best GUV quality and highest protein activity was obtained when electroformation was performed for 1 h at 10 Hz, 2.2 V_pp_ (peak-to-peak voltage, sine wave shape), followed by a detachment phase (detaching GUVs from Pt wires into the solution) of 30 min at 2–4 Hz, 2.2 V_pp_ (sine wave shape, Fig. 3b and Supplementary Video 1). After detachment, GUVs are collected by pipetting with a cut 1 ml micropipette tip and transferred directly to the imaging chamber, or stored refrigerated for up to a week in a microcentrifuge tube (Fig. 3a).

### GUV quality analysis — vesicle diameter and efficiency of protein reconstitution

**Figure 4:**
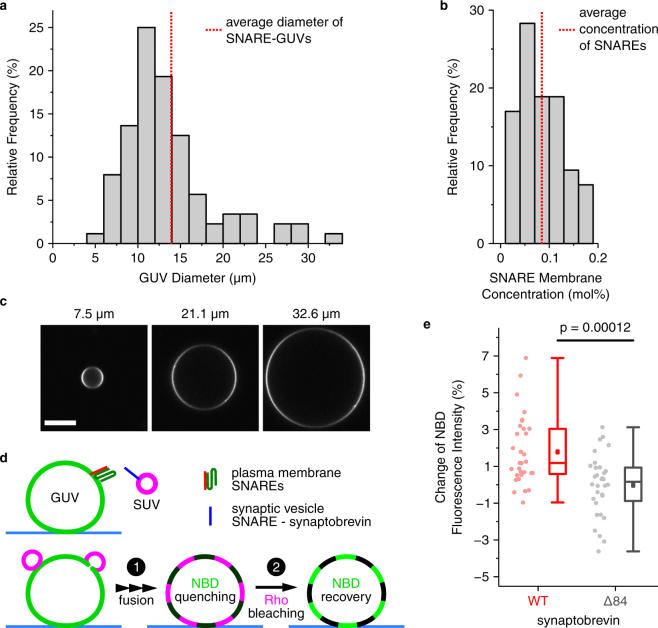
Characterization of SNARE-GUVs obtained with the Pt chamber. (**a**) Diameter distribution of GUVs obtained in a Pt chamber. The histogram was constructed from the diameters of N = 88 GUVs, with a bin width of 2 µm. Vertical dotted line indicates average diameter. (**b**) Histogram showing the distribution in the concentration of the SNARE proteins in the membrane of GUVs (N = 53, with a bin width of 0.03 mol%) obtained by comparing the fluorescence intensities of GUVs containing labelled proteins with those of GUVs containing known concentrations of labelled lipid (for details see Materials and Methods and ref.^18^) (**c**) Microscopy images showing examples of GUVs (stained with DiD) of different diameters obtained with the Pt chamber. Images were taken with the focal plane adjusted to the GUV equatorial plane. Scale bar 10 µm. Diameters are indicated above the images. (**d**) Cartoon showing the lipid mixing assay used for measuring membrane fusion of externally added SUVs with immobilised GUVs. Upon fusion (1) FRET between NBD and Rho results in NBD quenching. After bleaching of Rho (2), the NBD signal is recovered. (**e**) Lipid mixing experiment showing the fusogenic activity of SNARE proteins present on GUVs. NBD fluorescence intensity was measured before and after bleaching of the acceptor dye (Rho), with the changes after bleaching plotted as percentage change of total intensity. For control a synaptobrevin mutant (Δ84) was used which causes a block of fusion, with the vesicles arrested at the docked state. Boxes represent interquartile range, and whiskers below and above indicate full data range. Line in a box represents median and square point represents the mean. N = 32 for each experimental condition, unpaired *t*-test at α = 0.05.

Depending on the biological problem to be studied, the average diameter of GUVs may be critical. For instance, in some experiments handling and visualization of larger GUVs may be beneficial. The SNARE-GUVs prepared with a Pt chamber have diameters ranging from around 5 to 30 µm (Fig. 4a and c). Thus, the average diameter (13.5 µm, Fig. 4a) is substantially larger than that of the same GUVs prepared with ITO slides (5.8 µm)^18^. Another parameter critical for the assessment of GUV quality is the amount of protein incorporated in the membrane. In GUVs prepared with a Pt chamber we observe efficient protein incorporation by monitoring fluorescence intensity of Texas Red labelled proteins in the GUV membrane (Fig. 4b). By comparing these intensities with those of labelled lipid (for details see Materials and Methods and ref.^18^), the protein concentration in the membrane can be estimated (see histogram in Fig. 4b). Although the protein to lipid ratio showed some variability, there was no correlation with the size of the GUVs.

### GUV quality analysis — protein activity

SNARE proteins catalyse most membrane fusion reactions in eukaryotic cells^25^. Therefore, the best test for their activity upon membrane reconstitution and vesicle formation is to perform fusion assays^26^. Here we measured fusion using a lipid mixing assay^18,27^. In this experiment, immobilised GUVs containing labelled lipid NBD-PE as fluorescence donor and a stabilised complex of plasma membrane SNARE proteins^28^, were incubated with SUVs containing Lissamine Rhodamine-PE (Rho-PE) as fluorescence acceptor and the vesicular SNARE (schematic illustration in Fig. 4d). Upon SNARE-mediated membrane fusion, these two labels are in the same membrane and undergo Förster resonance energy transfer (FRET), causing quenching of NBD. If Rho is then bleached, a corresponding recovery of the NBD fluorescence intensity is observed (Fig. 4e, red). As a control for the specificity of this reaction, we used a synaptobrevin mutant (Δ84)^29^ that stops the fusion reaction at the docked state, preventing mixing of lipids and thus reducing FRET (Fig. 4e, grey).

## Discussion

Here we describe a convenient procedure for preparing proteo-GUVs containing SNARE-proteins of the presynaptic plasma membrane using in-house-built devices. The electroformation chamber described here is made from PTFE and thus can be cleaned with organic solvents. Moreover, the chamber allows for directly monitoring the formation of GUVs under a microscope. Additionally, the function generator used in this study can be easily programmed, allowing for testing of multiple electroformation protocols (a crucial step when establishing a protocol for proteo-GUV formation).

The protocol described here is convenient and avoids some of the problems associated with other methods. For instance, the procedures involving osmotic shock^6^ require repetitive drying-rehydration cycles, which are likely to be detrimental for maintaining membrane proteins in a functional state. Furthermore, gel-assisted swelling^30^ was reported to yield GUVs with altered mechanical properties^31^. Another possibility is to reconstitute proteins into the preformed GUVs with the aid of low concentrations of a detergent^10^, yet it requires extensive optimization of detergent type and concentration^3^, and it is very difficult to achieve efficient protein insertion while maintaining the GUVs intact. For special purposes, i.e. when membrane asymmetry is required, GUVs may be prepared with inkjet method^12^, however these technique requires a more specialised, and expensive equipment.

We conclude that our protocol offers a convenient method for the preparation of large GUVs containing moderate-high concentrations of membrane proteins. The yield of high-quality GUVs is comparably high, and only a single drying step is required, helping to preserve protein activity. For sensitive proteins, additional protection during the drying process may be necessary, for instance by adding disaccharides or ethylene glycol^3,32–34^.

## Materials and Methods

### Materials

Lipids were purchased from Avanti Polar Lipids: DOPC (1,2-dioleoyl-sn-glycero-3-phosphocholine), DOPE (1,2-dioleoyl-sn-glycero-3-phosphoethanolamine), DOPS (1,2-dioleoyl-sn-glycero-3-phospho-L-serine), 18:1 Biotinyl Cap PE (1,2-dioleoyl-sn-glycero-3-phosphoethanolamine-N-(cap biotinyl)), DOPS (1,2-dioleoyl-sn*-*glycero-3-phospho-L-serine), cholesterol (ovine wool), 18:1 NBD-PE (1,2-dioleoyl-sn-glycero-3-phosphoethanolamine-N-(7-nitro-2-1,3-benzoxadiazol-4-yl)), and 18:1 Liss Rho-PE (1,2-dioleoyl-sn-glycero-3-phosphoethanolamine-N-(lissamine rhodamine B sulfonyl)). Texas Red - coupled DHPE was purchased from Invitrogen. Lipophilic tracers — DiO and DiD, NeutrAvidin, biotinylated bovine serum albumin, and Texas Red maleimide (for protein labelling) were from Thermo Fisher Scientific. β-Casein (from bovine milk) was from Sigma-Aldrich, and Picodent Twinsil^®^ 22 (silicone glue) was from Picodent.

### Protein purification

Synaptic SNARE proteins: syntaxin-1A (183–288)^35^, SNAP-25 (cysteine free^36^ and S130C^37^), synaptobrevin-2 (wild type^38^ and Δ84 mutant^39^), and synaptobrevin-2 fragment (49–96)^28^; were derived from *Rattus norvegicus* and were expressed as pET28a constructs in *Escherichia coli* strain BL21 (DE3). Single proteins were purified via nickel-nitrilotriacetic acid affinity chromatography (Qiagen) and subsequent ion exchange chromatography on an Äkta system (GE Healthcare) with either MonoQ (syntaxin-1A and SNAP-25) or MonoS columns (synaptobrevin). For the proteins containing transmembrane region, i.e. syntaxin and synaptobrevin full length, buffer containing 1% CHAPS (3-[(3-Cholamidopropyl)dimethylammonio]-1-propanesulfonate, from Anatrace) or 1% octyl β-D-glucopyranoside (Glycon) was used, respectively. The assembly of the plasma membrane SNARE complex (consisting of syntaxin, SNAP-25, and synaptobrevin fragment 49–96) — a so called ΔN complex^28^ — was done by mixing the monomers overnight at 4 °C, followed by purification of the complex by ion exchange chromatography (MonoQ column) in a buffer containing CHAPS as described^28^. Fluorescence labelling of SNAP-25 (S130C) was carried out using Texas Red maleimide according to manufacturer’s instructions. Labelled ΔN complex was formed by replacing SNAP-25 with a S130C mutant labelled with Texas Red.

### Preparation of small unilamellar vesicles and fluorescent labelling of vesicles

Small unilamellar vesicles (SUVs) containing SNARE proteins (the plasma membrane SNARE complex or synaptobrevin) were prepared by co-micellization followed by size exclusion chromatography as described before^18^ with the following lipid composition: DOPC, DOPE, DOPS, and cholesterol at a molar ratio of 5:2:2:1. For immobilization of GUVs 1 mol% of DOPE was replaced with biotinyl-cap-PE, and for fluorescent labelling 1 mol% of DOPC was replaced with a membrane dye DiO or DiD. In case of lipid mixing experiments, GUVs contained 1.5 mol% NBD-PE whereas the SUVs were labelled with 1.5 mol% of Rho-PE. Protein was reconstituted at protein to lipid ratio of 1:1000 (SNARE complex in GUVs) or 1:500 (synaptobrevin in SUVs). Liposomes were formed in a buffer consisting of 20 mM HEPES/KOH pH 7.4, and 150 mM KCl, with a final lipid concentration of ∼0.7 mM determined according to ref.^40^.

### Preparation of giant unilamellar vesicles

GUVs containing SNARE proteins were prepared from vacuum-dried proteo-SUVs with the electroformation procedure using an in-house-built Pt electrode electroformation chamber (referred to as Pt chamber, see Fig. 2). The detailed GUV preparation protocol is described in the *Results* section.

Prior to use, the Pt chamber was cleaned by bath sonication (around 5–10 min) in ethanol and subsequently in chloroform. For sealing of the chamber, microscopy coverslips (25 mm in diameter) were used, that were first cleaned with ethanol and isopropanol, then coated with β-Casein (3 mg/ml, 5 min), and finally rinsed with water and dried.

### Microscopy imaging and data analysis

The formation of GUVs was directly monitored at low magnification in the electroformation chamber with an epifluorescence microscope. For visualization in higher magnification, GUVs were collected after the electroformation procedure and transferred to the imaging chamber containing a coverslip functionalised with biotinylated BSA and neutravidin^18^, and imaging buffer (20 mM HEPES/KOH pH 7.4, 150 mM KCl, 1 mM MgCl_2_, at least 1.5 × volume of the GUV solution to be added). GUVs were allowed to settle for around 30 min prior to imaging, resulting in surface attachment. Microscopy imaging was done with a Zeiss Axiovert 200 epifluorescence microscope or with a Zeiss LSM 780 confocal microscope.

The efficiency of protein reconstitution was determined as described in ref.^13^, following the detailed protocol described in ref.^18^, by comparing membrane fluorescence intensity of Texas Red labelled ΔN complex with those of known concentration of Texas Red labelled DHPE. Bulk lipid mixing experiments were performed essentially as described in ref.^18^. Image analysis was performed in Fiji^41^ with self-written scripts^18,42,43^.

### Data availability

The datasets generated during and/or analysed during the current study are available from the corresponding author on reasonable request.

## Electronic supplementary material

Supplementary Information

Supplementary Video 1
