# Supplementary material for: A convenient protocol for generating giant unilamellar vesicles containing SNARE proteins using electroformation
Source: Sci Rep. 2018 Jun 21;8:9422. doi: 10.1038/s41598-018-27456-4 (PMC6013450; doi:10.1038/s41598-018-27456-4)
Supplement: Supplementary file 1 — Supplementary Information [file 41598_2018_27456_MOESM1_ESM.pdf]

Supplementary Information

A convenient protocol for generating giant unilamellar vesicles containing SNARE proteins using electroformation

Agata Witkowska[1*], Lukasz Jablonski[1,2,3], and Reinhard Jahn[1*]

[1]Department of Neurobiology, Max-Planck-Institute for Biophysical Chemistry, Göttingen, Germany

[2]Present address: Institute for Auditory Neuroscience, University Medical Center Göttingen, Göttingen, Germany

[3]Present address: Auditory Neuroscience and Optogenetics Laboratory, German Primate Center, Göttingen, Germany

[*]to whom correspondence should be sent at:
Reinhard Jahn
e-mail: rjahn@gwdg.de
Agata Witkowska
e-mail: agata.witkowska@wp.eu

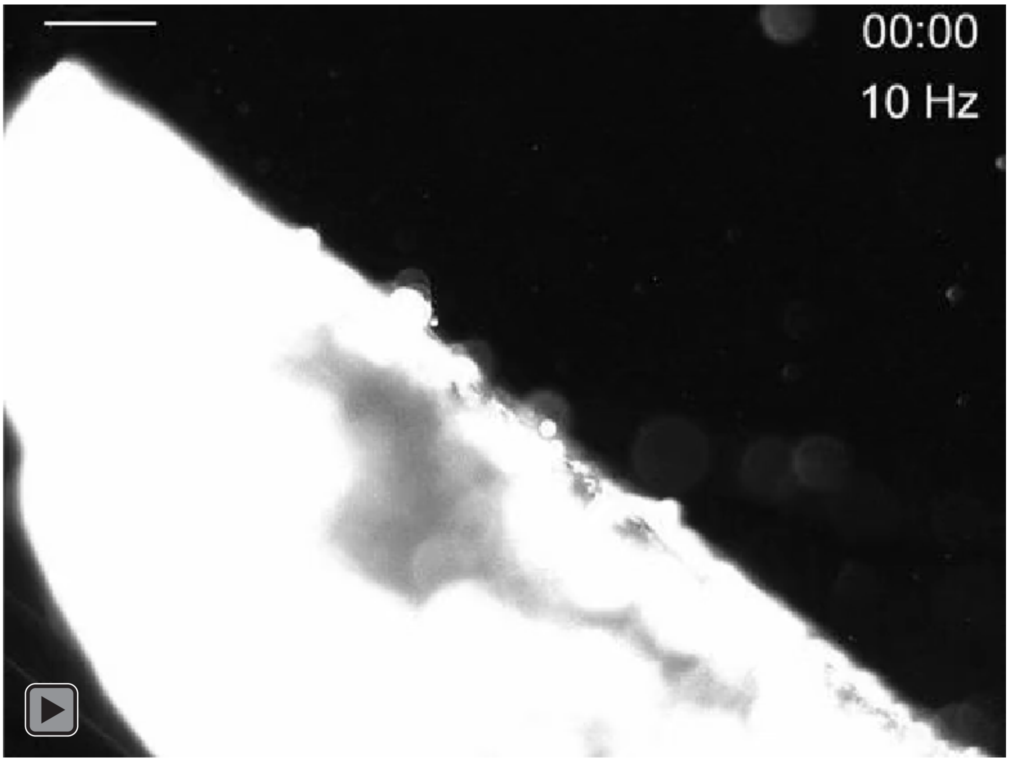

Supplementary Video 1. Video illustrating GUV formation in a Pt chamber. SNARE-GUVs (stained with DiO) forming during electroformation on Pt-wires. Time stamp (hours:minutes) and information about AC frequency are located in the top right corner. Scale bar (top left corner) 100 µm. Video is speeded up 300×.
